# Syndemic conditions and quality of life in the PISCIS Cohort of people living with HIV in Catalonia and the Balearic Islands: a cross sectional study

**DOI:** 10.1186/s12955-023-02120-2

**Published:** 2023-05-10

**Authors:** Jocelyn Mesías-Gazmuri, Cinta Folch, Jorge Palacio-Vieira, Andreu Bruguera, Laia Egea-Cortés, Carlos G. Forero, Juan Hernández, José M. Miró, Jordi Navarro, Melchor Riera, Joaquim Peraire, Lucía Alonso-García, Yesika Díaz, Jordi Casabona, Juliana Reyes-Urueña

**Affiliations:** 1 Centre of Epidemiological Studies of HIV/AIDS and STI of Catalonia (CEEISCAT), Health Department, Generalitat de Catalunya, Badalona, Spain; 2 Germans Trias I Pujol Research Institute (IGTP), Campus Can Ruti, Badalona, Spain; 3 PhD in Methodology of Biomedical Research and Public Health, Department of Pediatrics, Obstetrics and Gynecology, Preventive Medicine and Public Health, Universidad Autonoma de Barcelona, Badalona, Spain; 4 CIBER Epidemiología Y Salud Pública (CIBERESP), Madrid, Spain; 5 Department of Medicine. School of Medicine and Health Sciences, Universitat Internacional de Catalunya, Sant Cugat, Spain; 6 Grupo de Trabajo Sobre Tratamientos del VIH (gTt), Barcelona, Spain; 7 Infectious Diseases Service. Hospital Clínic-IDIBAPS. University of Barcelona, Barcelona, Spain; 8 CIBERINFEC, Instituto de Salud Carlos III, Madrid, Spain; 9 Infectious Diseases Department. Hospital, Universitari Vall d’Hebron, Barcelona, Spain; 10 Institut de Recerca Vall d’Hebron, Barcelona, Spain; 11 Hospital Son Espases, Palma de Mallorca, Spain; 12 Hospital Joan XXIII, IISPV, Universitat Rovira I Virgili, Tarragona, Spain; 13 Department of Pediatrics, Obstetrics and Gynecology and Preventive Medicine, Univ Autonoma de Barcelona, Badalona, Spain

**Keywords:** People living with HIV, health-related quality of life, HIV, Syndemic

## Abstract

**Background:**

People living with HIV (PLWH) face structural and psychosocial factors that affect health-related quality of life (HRQoL). We aimed to evaluate how syndemic conditions affected HRQoL in PLWH.

**Methods:**

A cross-sectional survey was conducted among 861 PLWH, to determine whether syndemic conditions (monthly income; sexual satisfaction; depressive symptoms; social role satisfaction; social isolation; cognitive function; nicotine dependence; perception of stigma) have an effect on HRQoL. A linear regression model and measures of Additive Interaction (AI) were used to determine the effects of syndemic conditions on HRQoL, controlling for other risk factors.

**Results:**

Overall, the most frequently observed were stigma perception (56.9%), poor cognitive function (50.6%) and the perception of social isolation (51.6%). The presence of depressive symptoms was the risk factor most associated with worse Physical Health (PH) (B 3.93, 2.71–5.15) and Mental Health (MH) (B 5.08, 3.81–6.34) in linear regression model. Specifically, an interaction was observed between poor cognitive function and poor satisfaction with social role on worse PH and MH (AI 2.08, 0.14–4.02; AI 2.69, 0.15–5.22, respectively); and low income and perception of stigma (AI 2.98, 0.26–5.71), low income and perception of social isolation (AI 2.79, 0.27–5.32), and low income and poor satisfaction with social role (AI 3.45, 0.99–5.91) on MH.

**Conclusion:**

These findings provide evidence that syndemic factors impact HRQoL. HIV prevention programs should screen and address co-occurring health problems to improve patient-centered health care and outcomes.

## Introduction

HIV continues to be a global public health problem with between 1.5 and 1.8 million new HIV infections occurring each year [1]. Recent data indicates that there are 37.7 million (30.2 million—45.1 million) PLWH worldwide [1], 151,387 in Spain [2] and 33,736 in Catalonia [3]. Combined antiretroviral therapy (cART) has changed HIV infection from a terminal illness to a chronic condition in countries where treatment is widely available [4, 5]. With the increasing availability of cART, PLWH enjoy almost the same life expectancy as the general population if the infection is diagnosed in an early stage and cART is prescribed right after diagnosis [6]. However, HRQoL can be adversely affected by a range of determinants such as cART side effects, chronicity of therapy, the ageing process, chronic inflammation and disease progression [7, 8], amongst others, these affect various life domains including the physical, mental, emotional, and social well-being domains. Consequently, improving and ensuring a good HRQoL among PLWH is increasingly important [9].

HRQoL is a multidisciplinary and multidimensional term that considers that an individual has their own perception of well-being, satisfaction and level of functioning [10, 11]. According to Urzúa et al., HRQoL is defined as the "level of well-being derived from the perception that a person has of various domains of her/his life, considering the impact that her/his state of health has on them" [12]. HRQoL is highly subjective, dynamic and unique to each individual [10], so ensuring standardized measurement of it is especially important to align HRQoL research priorities with the needs and values of patients, especially those with chronic illnesses such as HIV [11, 13].

Several previous studies have assessed the HRQoL of PLWH in different countries, which vary in terms of the associated factors and the dimensions of HRQoL evaluated [4, 7, 14–20]. Having symptoms of HIV, poor mental health, stigma, isolation and low social role were consistently reported to have adverse effects on HRQoL [14, 17]. For example, in a study conducted in Sweden, depression, self-stigma and social stigma were associated with lower HRQoL [18]. Moreover, it is known that the fear of discrimination, social stigma and low social support resulting in mental conflicts, isolation, depression and substance use [15, 19]. Similarly, another study carried out on PLWH in Uganda, all respondents identified low income as the main cause of worry and anxiety [4]. It has also been described that having a low income, has been associated with a lower satisfaction with sexual life, isolation, experiencing a fear of HIV transmission and not reporting a good HRQoL [21]. However, reports about the association of age, gender and antiretroviral therapy (ART) with HRQoL are inconsistent [7, 15–17, 20], probably, due to the discrepancies in the research methodologies used in those studies.

The syndemics theory posits that the co-occurrence and synergistic interaction of multiple adverse conditions or diseases within a population produce worse health outcomes than if each of the conditions was experienced separately [22, 23]. These problems can be biological, psychological, cultural, and environmental within the biopsychosocial concept of health [23] and are most likely to emerge under conditions of health inequality caused by poverty, low income, stigmatization, stress, or structural violence [22]. Singer's first findings indicated a combination of health issues (substance abuse, violence, and HIV/AIDS) and suggested that specific social factors such as poverty, discrimination, and exclusion from society were responsible for creating the environment in which these interactions took place [24]. In the United States a study among black HIV-positive individuals, which examined syndemic factors such as substance abuse, binge drinking, intimate partner violence, poor mental health, and risky sexual behavior, found that grouped syndemic conditions produced an effect that proportionally increased the risk of having an unsuppressed viral load, whereas when they were analyzed separately the risk was lower [25]. In line with these results, a study conducted among PLWH in Hong Kong, showed that those people who experienced co-occurring syndemic factors (stigma, social isolation and poor mental health) were more likely to engage in sexual risky behaviors, such as inconsistent condom use, compared to those who did not report syndemic conditions [26]. These psychosocial problems frequently occur in vulnerable populations, interact with each other, and the disease burden attributable to joint psychosocial problems exceeds the sum of the disease burden of these problems in isolation increasing the risk of poor clinical and health outcomes [27].

Although the existing evidence shows that the syndemic concurrent factors have a negative impact on the health of PLWH, very few studies have directly analyzed syndemic models to assess HRQoL among PLWH and most have used the cumulative approach to explain worse outcomes. Therefore, we propose that a psychosocial syndemic is an underlying mediator mechanism that negatively affects HRQoL of PLWH and clarifying the type of interaction has significant implications for intervention design. Consequently, this study is aimed at estimating the prevalence of syndemic conditions among PLWH and to describe the interaction pathways of these syndemic conditions on HRQoL.

## Methods

### Design and study population

Vive + is a cross sectional study conducted from October 2019 to March 2020 and nested in the PISCIS cohort, which is explained elsewhere [28]. Briefly, the PISCIS Cohort is a population-based cohort of PLWH from Catalonia and the Balearic Island (Spain) created in 1998 as a longitudinal, systematic, prospective, and multicenter study that provides population-based clinical, demographic and epidemiological data on patients with HIV infection.

The Vive + study included PLWH ≥ 18 years of age who signed the informed consent and who attended one of the units of the PISCIS cohort. Exclusion criteria were unable speak Spanish, unable to complete the questionnaire by themselves due to a mental disability or failure to agree to sign the consent form.

The sample size was calculated from the population under follow-up in PISCIS in 2017 (*n* = 14,190), for a maximum error of 5% and a significance level of 95% and taking into account an expected prevalence of depression and anxiety of 30% [29, 30], the total sample calculated was of 1,150 PLWH, assigned proportionally, according to the population on follow-up in each hospital. Women and people over 60 years of age were oversampled.

The study was approved by the Ethics Committee of the Germans Trias i Pujol Hospital (Nº PI-19–172) and by the ethics committee in each hospital where the recruitment was performed.

### Study procedures

Patients were invited to participate in the study at the PISCIS HIV units during a regular HIV visit by a trained peer. After signing the informed consent, those individuals who agreed to participate were provided with a piloted and adapted self-administered questionnaire in a portable electronic device (Tablet), which lasted approximately 40 min. The participants did not receive any financial incentives, as it was an opportunistic study, which did not receive external funding.

The data was stored in a central database located in the study coordinating center, following access management policies and the current data protection law [31], as well as the biomedical research law 14/2007 [32]. Patient information was anonymized and deidentified before the analysis.

Data from Vive + and from PISCIS were linked by a unique anonymized identifier assigned to each participant, which allowed researchers to extract HIV health related data (such as CD4 cell count and viral load).

### Questionnaire

The Vive + questionnaire collected information related to i). Sociodemographic and economic characteristics: gender (man, woman and transgender [given the small sample trans women and trans men are combined in this category]), age (< 39 years old; between 40 and 59 years old; > 60 years old), country of birth (Spain; outside Spain, educational level (≤ primary, or > primary [secondary, vocational training and/or university studies]), employment status (unpaid job: unemployed, student, housework; paid job: part-time or full-time employee, self-employed, retired, sick leave), monthly income (< 1000€; ≥ 1001€); ii) Psychosocial-sexual health characteristics: steady partner (person with whom they feel committed to above anyone else, includes partner, boyfriend or girlfriend, wife or husband), caring for others (responsibility to care for minors, adults or dependent elderly), perception of stigma and sexual satisfaction; ii). Lifestyle-related: nicotine dependence (Fageström test); polydrug use (used two or more illegal drugs in the last 12 months) for recreational purposes (Poppers [i.e., nitrates], Marijuana, Synthetic Cannabis, Ecstasy, MDMA, Amphetamines, Methamphetamine, Heroin, Mephedrone, Synthetic Stimulants, GHB, Ketamine, LSD, Cocaine, Crack) iii). HIV-related: serodisclosure, mode of transmission (people who inject drugs [PWID]; men who have sex with men [MSM]; men who have sex with women [MSW]; women who have sex with men [WSM]; other [those who did not know how they acquired HIV]; no answer), years living with HIV (< 13 years; ≧14 years); the last collected values of CD4 T-cell count (less than 200 cells/mm3, between 201 and 350 cells/mm3 and more than 351 cells/mm3 [33]) and viral load (detectable ≧ 50 copies/mL; undetectable < 50 copies/mL [34]).

HRQoL was measured through the 12-Item Short Form Health Survey v1 (SF-12v1), a freely distributed questionnaire [35], which consists of 12 items in 8 domains, with Likert-type response options that allow the generation of PH and MH summary scores, which are the two dimensions measure by the (SF-12v1) [36]. Each item has three to five options and the global score range from 0 to 100 points. The calculation of the scores is based on the Bidimensional Response Process Model Algorithm (BRP-12) [37], based on Item Response Theory [38]. The principal outcomes correspond to the dimensions that the SF-12 health questionnaire measures (PH and MH) and in this case, higher scores are indicative of poor PH and MH.

To assess the Perception of Stigma, the Neuro-QOL Item Bank Scale v1.0—Stigma [39], was used, a shortened version consisting of 8 questions with 5 response options on the Likert Scale. The final score ranges from 8 to 40 points. A higher perception of stigma is related to higher scores. To dichotomize the variable, the median was used as the cut-off point.

To assess the degree of physical dependence on nicotine, the Fageström Test [40] was used, which includes 6 items, generating a score between 0 and 10. It considers a standard classification, where scores greater than 7 indicate a high degree of nicotine dependence, but for the purposes of this study participants were classified as smokers (≥ 1 point) and non-smokers (0 point).

The presence of depressive symptoms in the last two weeks was evaluated through the Patient Health Questionnaire (PHQ-9), which is based on the criteria of the Diagnostic and Statistical Manual of Mental Disorders (DSM-IV) [41]. This instrument is made up of 9 items, each of which scores from 0 to 3, with a final score between 0 and 27 points. Scores of 5, 10, 15 and 20 represent the cut-off points to determine mild, moderate, moderately severe, and severe depressive symptoms, respectively. A score equal or less than 9 points was considered as an absence of symptoms or the presence of mild symptoms (coded 0) and a score higher than 9 points as an indicator of depressive symptoms (coded 1).

To measure satisfaction with participation in social roles and activities, and cognitive function, the Neuro-QOL Item Bank v1.1 and Neuro-QOL Item Bank v2.0 instruments were used, respectively [39, 42]. Both scales are self-reported measures that assess HRQoL and correspond to 8-item abbreviated measures, with 5 Likert-type response options ranging from never (1 point) to always (5 points). The final score ranges from 8 to 40 points, where higher scores indicate greater satisfaction with social roles and better cognitive function. The study uses the median as the cut-off point for both scales.

To assess social isolation the Patient-Reported Outcomes Measurement Information System (PROMIS®) Item Bank v2.0 (Social 8a) [43] instrument was used, which assesses the perceptions of being avoided or socially isolated, and consists of 8 questions, with 5 response options on a Likert-type scale. The results have been standardized with the population values, therefore 50 (standard deviation of 10) is the mean of the study population and in Vive + scores higher than the mean indicate a greater perception of social isolation.

Finally, sexual satisfaction was measured by asking: “In general, are you satisfied with your sexual life? And the possible responses were: (1) very satisfied, (2) satisfied, (3) unsatisfied, (4) very unsatisfied. Participants who answered (1) and (2) were coded “satisfied” (coded 0), and those who answered (3) and (4) were coded as “unsatisfied” (coded 1).

The syndemic factors considered were: 1) Monthly income; 2) Sexual satisfaction; 3) Presence of depressive symptoms in the last two weeks; 4) Satisfaction with social role; 5) Perception of social isolation; 6) Cognitive function; 7) Nicotine dependence; 8) Perception of stigma.

The dependent variables correspond to the dimensions that the SF12v1 health questionnaire measures, which are PH and MH.

### Statistical analysis

A general descriptive analysis was performed based on the calculation of PH and MH scores by sociodemographic and clinical characteristics, and syndemic factors. Frequencies (proportions) for qualitative variables and medians (interquartile range [IQR]) for quantitative variables were included.

For each outcome (domain), two linear regressions were performed. The first included each syndemic factor individually. The second contained those variables with a *p*-value < 0.2 in the bivariate analysis and considered as possible confounding factors. Before this, the presence of multicollinearity was tested by inspecting the correlation coefficients. For each model, the goodness of Fit was measured by the F test of global significance, the R2, the independence of the residuals (Durbin-Watson test), the graphical analysis of the residuals and the variance inflation factor [44].

Finally, to test the assertion that syndemic factors interact to affect HRQoL among PLWH, measures of additive interaction between the syndemic variables were computed. In linear regression, the regression coefficient of the product term reflects interaction as departure from additivity and it has been argued that this type of analysis better reflects biological interaction [45]. One of the main arguments for using the additive interaction approach instead of simple summation of syndemic factors is that the summation does not account for the complexity of interactions between different factors [46, 47]. The syndemic is not simply the co-occurrence of factors but is characterized by interaction between distinct factors that can amplify or diminish the effects of other factors [27]. In our study, we selected the Amount of Additive Interaction model because it is a robust and widely used approach to evaluate the extent to which the interaction of two factors explains differences in the variable of interest. The advantages of the Amount of Additive Interaction model are that it is easy to interpret, does not depend on the measurement scale of the factors or the variable of interest, is flexible and can be applied to different types of data and can be tailored to address specific questions, and can help identify factors that have significant interaction [48]. We opted for this model primarily because our variables were linear, making it a better fit for our data.

To perform the analysis, we ran independent linear regression models for each outcome and for each pair of syndemic variables, where we regressed their interactions adjusting by the potential confounder variables previously described. From these models, we obtain the Amount of Additive Interaction [48] with their corresponding 95% confidence intervals. We were unable to test for additive interaction beyond two exposures, as there is no consensus in the literature on a rigorous way to conduct this computation [47].

Therefore, in statistical terms, when entering two determinants, A and B, and a product term in a linear regression model, the regression formula of the outcome Y is [48]:

$$\mathrm Y=\mathrm\beta0+\mathrm\beta1\mathrm A+\mathrm\beta2\mathrm B+\mathrm\beta3\mathrm{AB}$$

From this equation, there are three possibilities for the regression coefficient of the product term:

i. If β3 = 0, the combined effect of A and B = β1 + β2—> exactly additivity—> no interaction as departure from additivity.
ii. If β3 < 0, the combined effect of A < B = β1 + β2—> less than additivity—> “negative” interaction as departure from additivity.
iii. If β3 > 0, the combined effect of A > B = β1 + β2—> more than additivity—> “positive” interaction as departure from additivity.

For this study, statistically significant Amount of Additive Interaction and different from 0, indicated the presence of interaction on the additive scale.

In the descriptive dataset, missing values are recorded under the name "No answer". The variable "years living with HIV" presented the highest number of missing values, with 10% of incomplete observations. In the linear regression model and in the interaction model, only variables with complete observations were included. Both models were adjusted for gender, age, country of birth, education level, and mode of transmission. For all the analyses, an alfa error level of 5% was considered. Data were analyzed with the Stata 16.1 program and R version 4.1.2.

## Results

From the total number of people who answered the survey (*n* = 1,060), 18.8% (*n* = 199) were excluded for having incomplete responses in the variables of interest, they were mainly men (55.3%), aged between 40–59 years (62.3%) and Spanish (66.3%). Of the people surveyed (*n* = 861), 81% (*n* = 697) were men, 15.8% (*n* = 136) were women, and 3.3% (*n* = 28) were transgender. Median age was 49 years-old (39–56), 66.7% (*n* = 574) were Spanish, 76.7% (*n* = 678) had an educational level higher than primary and 80.7% (*n* = 695) had a paid job. Regarding the mode of transmission, MSM (59.4%; *n* = 511), followed by PWID (18.7%; *n* = 161) and WSM (11.3%; *n* = 97) were the most prevalent groups (Table 1).

**Table 1:** Sociodemographic and clinical characteristics of people living with HIV by physical and mental health domains. Vive + study (2019–2020)

Variables	Overall		Physical Health		Mental Health	
	**N**	**%**	**Median**	**IQR**	**Median**	**IQR**
Sociodemographic characteristics						
Total	861	100	47.0	(42.8–53.6)	56.3	(48.9–66.0)
Gender						
Man	697	81.0	46.2	(42.5–51.6)	55.7	(48.9–65.7)
Woman	136	15.8	51.5	(45.1–57.1)	58.1	(49.4–67.5)
Transgender	28	3.3	56.2	(49.1–61.1)	61.1	(50.8–67.1)
No answer	8	0.8	48.6	(40.6–57.4)	65.1	(43.5–69.8)
Age						
< 39 years	228	26.5	45.2	(41.8–50.0)	58.2	(49.6–67.2)
40 – 59 years	499	58.0	47.0	(43.1–54.0)	56.1	(48.6–65.7)
> 60 years	134	15.6	51.4	(46.2–58.1)	54.7	(48.9–64.0)
Country of birth						
Spain	574	66.7	48.1	(43.4–54.7)	56.2	(48.9–65.5)
Outside Spain	287	33.3	45.9	(42.5–51.4)	56.8	(48.9–66.7)
Education level						
≦Primary	183	21.3	50.1	(45.5–58.1)	58.1	(47.8–66.7)
> Primary	678	76.7	46.2	(42.5–51.9)	55.7	(48.9–65.8)
No answer	17	1.7	48.8	(45.7–57.4)	59.8	(54.0–68.8)
Employment situation						
Paid job	695	80.7	46.5	(42.5–53.0)	55.6	(48.8–64.6)
Unpaid job	154	17.9	48.8	(44.5–56.2)	62.6	(49.4–68.2)
No answer	12	1.4	47.7	(41.3–51.8)	66.8	(46.5–69.8)
Stable couple						
Yes	488	56.7	46.2	(42.5–52.5)	55.4	(48.5–64.5)
No	363	42.2	48.1	(43.4–55.0)	58.1	(49.2–66.9)
No answer	10	1.2	47.0	(39.5–48.9)	57.3	(47.2–65.2)
Caring for others						
Yes	263	30.5	48.5	(43.4–55.3)	56.2	(47.3–66.0)
No	537	62.4	46.4	(42.6–52.2)	55.8	(48.9–65.9)
No answer	61	7.1	46.9	(42.5–54.6)	57.8	(49.7–66.6)
Mode of transmission ^a^						
PWID	161	18.7	50.8	(46.1–58.7)	61.4	(51.8–67.8)
MSM	511	59.4	45.9	(42.5–50.8)	55.4	(48.8–65.2)
MSW	58	6.7	47.0	(43.2–54.0)	54.1	(45.5–63.6)
WSM	97	11.3	50.2	(44.5–56.1)	57.2	(48.6–66.7)
Other	34	4.0	46.5	(41.5–54.9)	53.6	(40.7–60.7)
Be disclosure						
No	139	16.1	45.6	(42.0–51.7)	51.8	(45.5–61.6)
Yes	718	83.4	47.3	(43.2–54.2)	57.1	(49.2–66.5)
No answer	4	0.5	50.2	(48.0–53.6)	57.9	(49.6–64.7)
Clinical characteristics						
Years living with HIV						
< 13 years	387	45.0	45.7	(42.0–50.5)	56.1	(48.9–65.8)
≧14 years	388	45.0	49.5	(44.4–56.2)	57.0	(49.2–66.7)
No answer	86	10.0	46.6	(42.8–53.1)	54.6	(46.5–62.2)
CD4 (cel/ml) ^b^						
< 200	19	2.2	51.6	(46.1–57.5)	60.3	(49.6–66.4)
201–350	54	6.3	50.2	(47.6–58.8)	59.1	(50.8–67.2)
> 351	664	77.0	46.4	(42.5–52.7)	55.4	(47.9–65.7)
Not informed	124	14.4	48.2	(43.3–55.1)	58.4	(49.8–66.8)
Viral Load ^c^						
Detectable	53	6.2	48.1	(44.1–52.4)	60.6	(51.3–66.6)
Undetectable	665	77.2	46.5	(42.5–53.6)	55.6	(47.8–65.9)
Not informed	143	16.6	48.3	(43.6–54.8)	58.0	(49.2–66.5)

*IQR* Interquartile range

^a^ Mode of transmission: *PWID* People who inject drugs, *MSM* men who have sex with men, *MSW* men who have sex with women, *WSM* women who have sex with men

^b^ Lymphocyte count

^c^ Cut point: Viral load detectable > 51 copies

In respect of the clinical characteristics, 77% of the individuals had a CD4 count > 351 cells/ml, while 2.2% had < 200 cells/ml with a median of 14 (IQR: 6–24) years living with HIV and 77.2% (*n* = 665) had an undetectable viral load at the time of surveying (Table 1).

Regarding HRQoL domains, the median for MH was 56.3 (48.9–66.0) and the median for PH was 47.0 (42.8–53.6). Regarding PH those who had a median score greater than 50 points at the time of the survey, were women (51.5; 45.1–57.1), transgender people (56.2; 49.1–61.1), people over 60 years of age (51.4; 46.2–58.1), those with an education level ≤ primary (50.1; 45.5–58.1), those whose mode of transmission was through injecting drugs (50.8; 46.1–58.7) or WSM (50.2; 44.5–56.1), and those who had CD4 < 200 cells/ml (51.6; 46.1–57.5) and between 201–350 cells/ml (50.2; 47.6–58.8) (Table 1).

Regarding MH, those who had a median score greater than 60 points were transgender people (61.1; 50.8–67.1), those who reported having an unpaid job (62.6; 49.4–68.2), those whose mode of transmission was through injecting drugs (61.4; 51.8–67.8), those who had CD4 < 200 cells/ml (60.3; 49.6–66.4) and those who had a detectable viral load (60.6; 51.3–66.6) at the time of the survey (Table 1).

The most prevalent syndemic factors were perception of stigma (56.9%), perception of social isolation (51.6%) and poor cognitive function (49.4%) (Table 2).

**Table 2:** Physical health and mental health scores by syndemic factors and syndemic index score among people living with HIV. Vive + study (2019–2020)

Variables	Overall		Physical Health		Mental Health	
	**N**	**%**	**Median**	**IQR(a)**	**Median**	**IQR**
Monthly income						
< 1000€	375	43.6	50.2	(45.1–57.1)	59.4	(49.3–67.5)
≥ 1001€	486	56.4	45.4	(42.0–50.1)	54.7	(47.8–63.7)
Sexual satisfaction						
No	144	16.7	50.8	(46.4–57.1)	65.6	(54.8–69.4)
Yes	717	83.3	46.3	(42.5–52.4)	55.1	(47.6–64.4)
Presence of depressive symptoms in the last 2 weeks						
With symptoms	179	20.8	54.7	(49.2–59.8)	68.0	(64.6–70.5)
No symptoms or mild symptoms	682	79.2	45.9	(42.5–50.4)	53.7	(46.8–61.4)
Satisfaction with social role						
No	415	48.2	51.7	(45.6–57.0)	64.5	(55.8–68.5)
Yes	446	51.8	44.6	(41.7–48.4)	49.9	(44.9–57.1)
Perception of social isolation						
No	417	48.4	45.7	(42.2–50.0)	50.8	(44.6–58.4)
Yes	444	51.6	49.2	(43.6–55.7)	62.7	(54.3–68.1)
Cognitive function						
Poor	425	49.4	50.5	(44.9–57.2)	64.0	(54.8–68.3)
Good	436	50.6	45.1	(42.0–48.9)	51.1	(44.9–58.5)
Nicotine dependence						
No	484	56.2	46.5	(42.5–53.6)	54.9	(47.8–64.8)
Yes	377	43.8	48.1	(43.4–53.6)	58.5	(49.2–66.9)
Perception of stigma						
No	371	43.1	46.0	(42.0–50.2)	52.1	(44.9–60.4)
Yes	490	56.9	48.4	(43.6–55.5)	59.4	(51.7–67.4)

*IQR* Interquartile range

Table 3 presents the association between each syndemic condition with the two HRQoL domain outcomes. In the unadjusted model, all syndemic conditions, except nicotine dependence in PH, were associated individually with a higher impairment in both domains (PH and MH) (Table 3).

**Table 3:** Simple linear regression model of syndemic factors and syndemic index by physical and mental health domains of people living with HIV. Vive + study (2019–2020)

Variables	Physical Health			Mental Health		
	**B**	**95% CI**^a^	***P* value**	**B**	**95% CI**	***P* value**
Syndemic factors						
Monthly income						
< 1000€	4.82	(3.83–5.82)	0.000	3.22	(1.82–4.63)	0.000
Sexual satisfaction						
Unsatisfied	3.92	(2.55–5.28)	0.000	7.10	(5.28–8.93)	0.000
Presence of depressive symptoms in the last 2 weeks						
With symptoms	7.64	(6.46–8.81)	0.000	13.32	(11.83–14.81)	0.000
Satisfaction with social role						
No	5.56	(4.59–6.53)	0.000	10.82	(9.61–12.03)	0.000
Perception of social isolation						
Yes	3.10	(2.08–4.11)	0.000	9.60	(8.34–10.85)	0.000
Cognitive function						
Poor	4.83	(3.84–5.82)	0.000	9.62	(8.36–10.87)	0.000
Nicotine dependence						
Dependence	0.72	(0.33-.1.76)	0.180	2.18	(0.76–3.59)	0.003
Perception of stigma						
Yes	2.79	(1.76–3.82)	0.000	6.67	(5.32–8.02)	0.000

^a^ *CI 95%* confidence interval

Table 4 presents results from multivariate analysis confounder adjusted. In particular, monthly income (B 2.33; CI95% 1.29–3.17), presence of depressive symptoms in the last 2 weeks (B 3.93; CI95% 2.71–5.15), satisfaction with social role (B 2.51; CI95% 1.50–3.51), cognitive function (B 1.60; CI95% 0.60–2.61) and perception of stigma (B 1.26; CI95% 0.34–2.19) were significantly associated with the PH domain. While sexual satisfaction (B 2.49; CI95% 1.01–3.96), presence of depressive symptoms in the last 2 weeks (B 6.93; CI95% 5.40–8.46), satisfaction with social role (B 5.08; CI95% 3.81–6.34), perception of social isolation (B 2.91; CI95% 1.62–4.20), cognitive function (B 3.13; CI95% 1.87–4.40) and perception of stigma (B 1.87; CI95% 0.71–3.03) were associated with MH.

**Table 4:** Adjusted linear regression of the syndemic factors and syndemic index by physical and mental health domains of people living with HIV. Vive + study (2019–2020)

Variables	Physical Health ^a^			Mental Health ^a^		
	**B**	**95% CI** ^b^	***P* value**	**B**	**95% CI**	***P* value**
Syndemic factors						
Monthly income						
< 1000€	2.23	(1.29–3.17)	0.000	0.40	(-1.57–0.78)	0.507
Sexual satisfaction						
Unsatisfied	0.89	(-0.28–2.07)	0.136	2.49	(1.01–3.96)	0.001
Presence of depressive symptoms in the last 2 weeks						
With symptoms	3.93	(2.71–5.15)	0.000	6.93	(5.40–8.46)	0.000
Satisfaction with social role						
No social role	2.51	(1.50–3.51)	0.000	5.08	(3.81–6.34)	0.000
Perception of social isolation						
Yes	0.29	(-0.60–0.74)	0.581	2.91	(1.62–4.20)	0.000
Cognitive function						
Poor	1.60	(0.60–2.61)	0.000	3.13	(1.87–4.40)	0.000
Nicotine dependence						
Dependence		-		0.84	(-0.26–1.95)	0.133
Perception of stigma						
Yes	1.26	(0.34–2.19)	0.008	1.87	(0.71–3.03)	0.002

^a^ The models were adjusted for: gender, age, country of birth, educational level, and mode of transmission

^b^ *CI 95%* confidence interval

There was evidence for additive interaction (i.e., synergism) between some of the syndemic factors (Table 5). In particular, a positive interaction was detected between satisfaction with social role and cognitive function (2.08, 0.14–4.02) for a worse PH; and between monthly income with satisfaction with social role, perception of social isolation, and perception of stigma, (3.45, 0.99–5.91; 2.79, 0.27–5.32; and 2.98, 0.26–5.71, respectively). For a worse MH, an interaction was found between satisfaction with social role and cognitive function (2.69, 0.15–5.22). Drawing on the original conceptualization of syndemic theory, these results would indicate a positive interaction or also an excessive burden of adversity for PH or MH, given that the combinations of previously described syndemic factors are greater than their individual burden. The rest of the factors studied did not reveal statistically significant synergies.

**Table 5:** Additive interaction of syndemic exposures on physical and mental health domains of people living with HIV. Vive + study (2019–2020)

		Physical Health ^**a**^		Mental Health ^**a**^	
Syndemic factors		AI	95% CI ^**b**^	AI	95% CI ^**b**^
Income	Sexual S	-1.05	(-3.52–1.42)	-0.09	(-3.69–3.51)
Income	Depression	-0.47	(-2.75–1.81)	1.13	(-2.03–4.28)
Income	Social R	0.07	(1.37–1.87)	3.45	(0.99–5.91)
Income	Isolation	1.66	(-0.18–3.51)	2.79	(0.27–5.32)
Income	Cognitive F	1.06	(-0.75–2.88)	0.78	(-1.76–3.33)
Income	Nicotine D	0.81	(-1.09–2.71)	1.32	(-1.50–4.13)
Income	Stigma	1.54	(-0.33–3.42)	2.98	(0.26–5.71)
Sexual S	Depression	-2.38	(-4.92–0.17)	-0.14	(-3.58–3.29)
Sexual S	Social R	-0.03	(-2.59–2.53)	-0.02	(-3.42–3.38)
Sexual S	Isolation	1.33	(-1.35–4.01)	-0.79	(-4.34–2.76)
Sexual S	Cognitive F	1.40	(-0.63–3.42)	1.32	(-2.13–4.76)
Sexual S	Nicotine D	0.51	(-1.37–2.39)	0.00	(-3.62–3.62)
Sexual S	Stigma	1.56	(-0.41–3.53)	-3.21	(-7.18–0.75)
Depression	Social R	-1.12	(-4.12–1.89)	-3.35	(-7.23–0.53)
Depression	Isolation	1.31	(-1.29–3.81)	-3.03	(-6.98–0.92)
Depression	Cognitive F	-1.91	(-4.93–1.11)	-4.38	(-8.34–0.41)
Depression	Nicotine D	-0.47	(-2.75–1.81)	0.12	(-2.78–3.12)
Depression	Stigma	1.31	(-1.19–3.81)	0.56	(-2.80–3.91)
Social R	Isolation	0.32	(-1.65–2.29)	2.37	(-0.16–4.91)
Social R	Cognitive F	2.08	(0.14–4.02)	2.69	(0.15–5.22)
Social R	Nicotine D	0.94	(-0.87–2.76)	-0.35	(-2.79–2.09)
Social R	Stigma	0.77	(-1.09–2.64)	-0.01	(-2.49–2.47)
Isolation	Cognitive F	1.40	(-0.63–3.42)	2.08	(-0.57–4.73)
Isolation	Nicotine D	0.94	(-0.87–2.76)	1.01	(-1.51–3.53)
Isolation	Stigma	1.56	(-0.41–3.53)	0.13	(-2.50–2.77)
Cognitive F	Nicotine D	0.80	(-1.04–2.64)	1.07	(-1.44–3.59)
Cognitive F	Stigma	1.68	(-0.20–3.56)	0.68	(-1.86–3.22)
Nicotine D	Stigma	0.79	(-1.12–2.71)	1.58	(-1.15–4.30)

*AI* Additive interaction, *Income* Monthly income, *Sexual S* Sexual satisfaction, *Depression* Presence of depressive symptoms, *Social R.* Satisfaction with social role, *Isolation* Perception of social isolation, *Cognitive F.* Cognitive function, *Nicotine D.* Nicotine dependence, *Stigma* Perception of stigma

^a^ The models were adjusted for: gender, age, country of birth, educational level, and mode of transmission

^b^ *CI 95%* confidence interval

## Discussion

To our knowledge, this is the first study that analyzes HRQoL in PLWH using a syndemic approach and syndemic factors interaction in Spain. Our findings describe a synergic interaction between psychosocial and structural factors that negatively impact on HRQoL in PLWH. With the exception of sexual satisfaction and perceptions of social isolation in the PH domain, and low income and nicotine dependence in the MH domain, the results were associated with worse PH and MH. The prevalence of syndemic factors was high, being the most prevalent stigma, poor cognitive function, and perception of social isolation. The presence of depressive symptoms was identified as the highest risk factor for a worse outcome in both PH and MH domains. In addition, an interaction was found between poor cognitive function and poor satisfaction with social role for PH and MH, as well as an interaction between low income, perception of stigma, perception of social isolation, and poor satisfaction with social role in MH. These findings highlight the importance of considering syndemic conditions as determinants of HRQoL, which means it is of major importance to include them when designing patient-centered strategies to improve outcomes in health among PLWH.

In accordance with previous studies [49–51], we found a high prevalence of syndemic factors. The association between a high prevalence of syndemic factors and a worse HRQoL was previously described by Oliveira et al. [49], who observed that the presence of syndemic factors led to poorer HRQoL scores in the psychological, social, independence and environmental domains. Similarly, other studies [4, 17, 26, 52] have found an association between HRQoL and perception of stigma, a psychological symptom that affects PLWH greatly [53]. In this regard, Chan et al. [26], concludes that HIV-related stigma and discrimination positively predicts the number of psychosocial syndemic problems, given that a cluster of syndemic conditions may develop as a result of negative life experiences.

We found few studies evaluating cognitive function in PLWH and they are mainly focused on older people [54] or people with auto degenerative diseases [55]. Similar to our study, the reported prevalence of cognitive impairment in PLWH varies between 30 and 60% [56, 57] and cognitive dysfunction is more prevalent among older adults living with HIV [54, 55], which would be expected, given that cognitive impairment is known to increase with aging. Greater age has also been associated with fewer social interactions and a smaller social network [57], this data is in agreement with our study. PLWH are likely to disengage from social interaction and withdraw from their community, resulting in social isolation [58, 59]. Furthermore, secrecy of HIV status disclosure often leads to loneliness and isolation [51]. These psychosocial problems frequently co-occur among PLWH, potentially compounding the risk of poor clinical outcomes, contributing to worse HRQoL and potentially promoting syndemic interactions.

Although depressive symptoms were the syndemic factor with a stronger association with a worse HRQoL, most syndemic conditions were associated with an impairment in HRQoL in both domains (PH and MH), even after adjustment for several confounding variables. A range of factors could be related to high prevalence of depressive symptoms among PLWH, including those related to living with a chronic disease [14, 60], low income or living in poverty [4, 22], stigma and discrimination [26], feelings of loneliness [30, 61] or fear of disclosure [61, 62]. Whatever the drivers of depression, early identification and treatment are of great importance, as depression has been associated with poor adherence to antiretroviral therapy (ART) [63–65]. Therefore, depression represents a great challenge for health care workers and public health specialists, as treating depression pharmacologically might not ensure swift recovery when symptoms stem from problems in the patient’s social life and coexist in a syndemic context. Thus, training health care professionals in mental health is needed to assure a holistic approach to detect depressive symptom in early stages to minimize its impact in HRQoL.

The relationship established between dissatisfaction with social role and poor cognitive function in PLWH is a complex interaction that produces more serious effects than those resulting from each factor separately. In particular, dissatisfaction with social role and poor cognitive function combine to affect real-life activities in PLWH, including adherence to ART [66, 67], performance of tasks important to social function [68, 69], emotional and social well-being [69], to cause unhealthy behaviors and medical complications [67]. Furthermore, this interaction can create a vicious cycle, in which poor mental and physical health can contribute to further social isolation and discrimination, which in turn can worsen poor cognitive function and dissatisfaction with social role [70, 71]. Tozzi et al. concluded, that HRQoL is influenced by cognitive impairment and by the ability to engage in activities of everyday living [72]. In turn, it has been described that stress resulting from stigma and dissatisfaction with social role can increase cortisol levels in the body, which at the same time can negatively affect the immune system and worsen physical health [73, 74]. These findings highlight the importance of addressing psychosocial factors in the care and treatment of PLWH, not only to improve their psychological well-being, but also to improve their long-term physical health.

Although at the individual level, low income did not show a relationship with worse MH, when combined variables were evaluated, there was evidence of an interaction between this variable and stigma, isolation, and dissatisfaction with the social role. There is little evidence regarding the mechanisms that link these factors, however, it is known that HIV is a disease that is rooted in social and economic inequality [75]. In this context, Kang et al., describe that poverty is associated with worse MH functioning and stigma of PLWH, and suggest that interventions that are integrated with economic livelihood programs [76]. In addition, the stigma associated with HIV can affect self-esteem and consequently the ability to seek employment, increase income or maintain regular employment [77, 78]. On the other hand, the lack of economic resources can limit access to adequate treatments, adherence to ART and healthy eating, which can increase the risk of MH problems [76] and contribute to problems such as anxiety and depression [79]. Also, stigma and discrimination can make it difficult to build healthy social and emotional relationships, which can increase isolation, dissatisfaction with social roles, and emotional stress [80]. Prior studies of livelihood interventions for PLWH have similarly found that improved economic standing was coupled with “social reintegration and reversal of status loss” [78, 81]. According to the syndemic theory [27], addressing one of these factors reduces costs, complexity, and overall intervention times, positively affecting the HRQoL of PLWH.

This study has limitations that should be considered. Firstly, our data came from a convenience sample in two Spanish autonomous communities, therefore, they cannot be extrapolated to the rest of the PLWH in Spain. In addition, the recruitment of participants was done in outpatient clinics (HIV care units), excluding hospitalized patients and those who cannot or were not attending clinics, however, the representative sample of PLWH was sufficiently powered to identify the variables being studied and showed important results given the scarcity of quantitative research that assess HRQoL in PLWH with a syndemic approach. Secondly, the study was based on self-reported data which may lead to under-reporting of certain responses, or measurement or social desirability biases, and lead to underestimating some sensitive behaviors. However, the high response rate and the help of a peer who created a confidential environment, make us confident in the validity of the responses, which can reduce the above-described bias, as has been shown in other studies [82]. In addition, at the time of the study and data collection, the comorbidity variable was not collected. Therefore, future studies should include this variable, since it is a key factor influencing HRQoL. Third, given the cross-sectional design, the direction of the associations between syndemic factors and HRQoL cannot be established, and it is not possible to discard reverse causality, for example, that worse HRQoL in the mental domain could be the cause of depression and social isolation and not conversely [83]. It is for this reason that Vive + will be performed periodically, as longitudinal assessment will be better to describe the onset and trajectory of syndemic factors as well as their impact on HRQoL. Finally, it is important to acknowledge that we conducted a secondary analysis of data collected for a HRQoL in PLWH. Therefore, syndemic theory did not influence the research design, data collection instrument or identification of constructs. The literature demonstrates that most contemporary syndemic studies are based on secondary analyses [25, 26, 49]. Future studies are urgently needed to advance the methodological basis of syndemic analysis and further elucidate relationships between syndemic factors and HRQoL in PLWH.

## Conclusion

In summary, this study highlights the impact of syndemic factors on HRQoL in PLWH. While the approach used in this study only examined the impact of two syndemic factors at a time on HRQoL, the results suggest that certain characteristics can overlap and pointed out a group of PLWH with higher vulnerability. Harmful interactions are often overlooked, particularly in adverse socioeconomic and behavioral circumstances, which likely enhances syndemic clustering. Therefore, addressing multiple syndemic factors simultaneously is necessary to improve HRQoL outcomes in PLWH. Further research could help better understand the interaction of these factors and their role in promoting disease clustering at the population level, enabling a more holistic approach on the clinical management of PLWH.

Moreover, integrating HRQoL assessment into clinical practice will significantly advance public health because, at the individual level, this can predict behaviors that negatively affect health and at population level, it will help identify the weaknesses in the health system. This change of strategy is imperative and highlights the urgent need to reshape the conventional understanding of diseases as separate and independent from other diseases and to take into account the social contexts in which diseases are found. A syndemic-based perspective offers a holistic approach to address diseases, taking into consideration psychosocial factors or other adverse health conditions, as a set of conditions that threaten HRQoL.

## Data Availability

The study protocol is available from Dr. Juliana Reyes-Urueña (e-mail: jmreyes@iconcologia.net). Statistical code for the analysis can be requested from Yesika Diáz, Sergio Moreno, and Jordi Aceiton (ydiazr@iconcologia.net, smorenof@iconcologia.net, jaceiton@igtp.cat). The data for this study is available at the Centre for Epidemiological Studies of Sexually Transmitted Diseases and HIV/AIDS in Catalonia (CEEISCAT), the coordinating centre of the PISCIS cohort and from each of the collaborating hospitals upon request via https://pisciscohort.org/contacte/.

## Abbreviations

AI: Additive interaction
ART: Antiretroviral therapy
BRP: Bidimensional Response Model
cART: Combined antiretroviral therapy
DSM-IV: Diagnostic and Statistical Manual of Mental Disorders
HRQoL: Health-related quality of life
IQR: Interquartile range
MH: Mental Health
MSM: Men who have sex with men
MSW: Men who have sex with women
PH: Physical Health
PHQ-9: Patient Health Questionnaire
PLWH: People living with HIV
PROMIS: Patient-Reported Outcomes Measurement Information System
PWID: People who inject drugs
SF-12: 12-Item Short Health Survey
WSM: Women who have sex with men

## References

[1] Programa Conjunto de las Naciones Unidas sobre el VIH/SIDA. Hoja informativa — Últimas estadísticas sobre el estado de la epidemia de sida. Onusida. 2021. (Accesed 12 Jul 2022). Available from: Available from: https://www.unaids.org/es/resources/fact-sheet

[2] Unidad de vigilància del VIH, ITS y hepatitis. Actualización del Continua de Atención del VIH en España, 2017–2019. Madrid: Centro Nacional de Epidemiología - Instituto de Salud Carlos III / Plan Nacional sobre el SIDA – Dirección General de Salud Pública; 2020. Available from: https://www.sanidad.gob.es/ca/ciudadanos/enfLesiones/enfTransmisibles/sida/vigilancia/ESTIMACION_DEL_CONTINUO_DE_ATENCIoN_DEL_VIH_EN_ESPAnA_Nov2020.pdf.

[3] Centre d’Estudis Epidemiològics sobre les Infeccions Transmissió Sexual i Sida de Catalunya (CEEISCAT). Memòria de resultats de la Cohort Poblacional de VIH Catalano Balear, PISCIS 1998–2018. Badalona; 2018. (Accesed 30 Aug 2021). Available from: www.ceeiscat.cat

[4] Mutabazi-Mwesigire D, Seeley J, Faith M, Katamba A (2014). Perceptions of quality of life among Ugandan patients living with HIV: a qualitative study. BMC Public Health.

[5] Cooper V, Clatworthy J, Harding R, Whetham J (2017). Emerge Consortium Measuring quality of life among people living with HIV: a systematic review of reviews. Health Qual Life Outcomes..

[6] Nakagawa F, Lodwick RK, Smith CJ, Smith R, Cambiano V, Lundgren JD (2012). Projected life expectancy of people with HIV according to timing of diagnosis. AIDS.

[7] Park-Wyllie LY, Strike CS, Antoniou T, Bayoumi AM (2007). Adverse quality of life consequences of antiretroviral medications. AIDS Care.

[8] Slim J, Saling CF. A review of management of inflammation in the HIV population. Biomed Res Int. 2016:3420638. 10.1155/2016/3420638. 10.1155/2016/3420638 PMC5059528 27766258

[9] Popping S, Kall M, Nichols BE, Stempher E, Versteegh L, AMC van de Vijver D (2021). Quality of life among people living with HIV in England and the Netherlands: a population-based study. Lancet Reg Health Eur..

[10] Urzúa MA, Caqueo-Urízar A (2012). Calidad de vida: una revisión teórica del concepto. Ter Psicol.

[11] Clayson DJ, Wild DJ, Quarterman P, Duprat-Lomon I, Kubin M, Coons SJ (2006). A comparative review of health-related quality-of-life measures for use in HIV/AIDS clinical trials. Pharmacoeconomics.

[12] Urzúa MA (2010). Calidad de vida relacionada con la salud: elementos conceptuales. Rev Med Chil.

[13] Bakas T, McLennon SM, Carpenter JS, Buelow JM, Otte JL, Hanna KM (2012). Systematic review of health-related quality of life models. Health Qual Life Outcomes.

[14] Miners A, Phillips A, Kreif N, Rodger A, Speakman A, Ficher M (2014). Health-related quality-of-life of people with HIV in the era of combination antiretroviral treatment: a cross-sectional comparison with the general population. Lancet HIV.

[15] George S, Bergin C, Clarke S, Courtney G, Codd MB (2016). Health-related quality of life and associated factors in people with HIV: An Irish cohort study. Health Qual Life Outcomes.

[16] Carter A, Loutfy M, de Pokomandy A, Colley G, Zhang W, Sereda P (2018). Health-related quality-of-life and receipt of women-centered HIV care among women living with HIV in Canada. Women Health.

[17] Castro R, De Boni RB, Luz PM, Velasque L, Lopes LV, Medina-Lara A (2019). Health-related quality of life assessment among people living with HIV in Rio de Janeiro, Brazil: a cross-sectional study. Qual Life Res.

[18] Zeluf-Anderson G, Eriksson LE, Schönnesson LN, Höijer J, Månehall P, Ekström AM (2019). Beyond viral suppression: the quality of life of people living with HIV in Sweden. AIDS Care.

[19] Mahalakshmy T, Premarajan KC, Hamide A (2011). Quality of life and its determinants in people living with human immunodeficiency virus infection in Puducherry, India. Indian J Community Med.

[20] Pérez IR, Olry De Labry Lima A, Del Castillo LS, Baño JR, Ruz MÁL, Del Arco Jimenez A. No differences in quality of life between men and women undergoing HIV antiretroviral treatment. Impact of demographic, clinical and psychosocial factors. AIDS Care*.* 2009;21(8):943–52. 10.1080/09540120802612840. 10.1080/09540120802612840 20024750

[21] Peyre M, Gauchet A, Bissuel F, Blanc M, Boibieux A, Cotte L (2019). Satisfaction with sexual life in people living with HIV/AIDS: the persistent weight of the fear of transmission. AIDS Care.

[22] Singer M, Bulled N, Ostrach B (2017). Syndemics and the biosocial conception of health. Lancet.

[23] Singer M, Clair S (2003). Syndemics and public health: reconceptualizing disease in bio-social context. Med Anthropol Q.

[24] Singer M (1996). A dose of drugs, a touch of violence, a case of AIDS: Conceptualizing the SAVA Syndemic. Free Inq Creat Sociol.

[25] Sullivan KA, Messer LC, Quinlivan EB (2015). Substance abuse, violence, and HIV/AIDS (SAVA) syndemic effects on viral suppression among HIV positive women of color. AIDS Patient Care STDS.

[26] Chan RCH, Operario D, Mak WWS (2020). Effects of HIV-related discrimination on psychosocial syndemics and sexual risk behavior among people living with HIV. Int J Environ Res Public Health.

[27] Singer M, Bulled N, Ostrach B (2020). Whither syndemics?: Trends in syndemics research, a review 2015–2019. Glob Public Health.

[28] Jaén Á, Casabona J, Esteve A, Miró JM, Tural C, Ferrer E (2005). Características clinicoepidemiológicas y tendencias en el tratamiento antirretroviral de una cohorte de pacientes con infección por el virus de la inmunodeficiencia humana. Cohorte PISCIS Med Clin.

[29] Heras S, Astorga E, Herencias A, Cervigon R, Galvez J (2018). Prevalencia y tipos de trastornos psiquiátricos en la población VIH de un Centro de Salud. Rev Multidiscip del Sida.

[30] Slot M, Sodemann M, Gabel C, Holmskov J, Laursen T, Rodkjaer L (2015). Factors associated with risk of depression and relevant predictors of screening for depression in clinical practice: a cross-sectional study among HIV-infected individuals in Denmark. HIV Med.

[31] Ley Orgánica 3/2018, De 5 De Diciembre, De Protección De Datos Personales Y Garantía De Los Derechos Digitales. Protección datos Pers*.* 2020;145–252. (Accesed 20 Nov 2021). Available from: https://www.boe.es/eli/es/lo/2018/12/05/3.

[32] Ley 14/2007, de 3 de julio, de Investigación biomédica. 2007; 28826–48. (Accesed 20 Nov 2021). Available from: https://www.boe.es/buscar/doc.php?id=BOE-A-2007-12945.

[33] Ford N, Meintjes G, Vitoria M, Greene G, Chiller T (2017). The evolving role of CD4 cell counts in HIV care. Curr Opin HIV AIDS.

[34] Panel on Antiretroviral Guidelines for Adults and Adolescents. Guidelines for the Use of Antiretroviral Agents in Adults and Adolescents with HIV. Department of Health and Human Services. 2021. (Accesed 14 Nov 2021). Available from: http://hivinfo.nih.gov.

[35] Rand Corporation. 12-Item Short Form Survey (SF-12) | RAND. (Accesed 13 Jul 2021). Available from: https://www.rand.org/health-care/surveys_tools/mos/12-item-short-form.html.

[36] Schmidt S, Vilagut G, Garin O, Cunillera O, Tresserras R, Brugulat P (2012). Normas de referencia para el Cuestionario de Salud SF-12 versión 2 basadas en población general de Cataluña. Rev Clin.

[37] Forero CG, Vilagut G, Alonso J. *Obtención de puntuaciones en cuestionario SF-12 mediante modelos RT multidimensionales.* In: XXIX Reunión Científica de la SEE y XIV Congreso SESPAS. Madrid, Spain; 2011.

[38] Forero CG, Vilagut G, Adroher ND, Alonso J (2013). Multidimensional item response theory models yielded good fit and reliable scores for the Short Form-12 questionnaire. J Clin Epidemiol.

[39] National Institute of Neurological Disorders and Stroke (NINDS). User Manual Quality of Life in Neurological Disorders (Neuro-QoL) Measures. 2015, version 2.0. (Accesed 25 Jul 2021). Available from: https://www.sralab.org/sites/default/files/2017-06/Neuro-QOL_User_Manual_v2_24Mar2015.pdf.

[40] Becoña E, Vázquez FL (1998). The Fagerström test for nicotine dependence in a Spanish sample. Psychol Rep.

[41] Kroenke K, Spitzer RL, Williams JB (2001). The PHQ-9: validity of a brief depression severity measure. J Gen Intern Med.

[42] Cella D, Lai JS, Nowinski CJ, Victorson D, Peterman A, Miller D (2012). Neuro-QOL: brief measures of health-related quality of life for clinical research in neurology. Neurology.

[43] Fox RS, Peipert JD, Vera-Llonch M, Phillips G, Cella D (2020). PROMIS® and Neuro-QoLTM measures are valid measures of health-related quality of life among patients with familial chylomicronemia syndrome. Expert Rev Cardiovasc Ther.

[44] Akinwande MO, Dikko HG, Samson A (2015). Variance inflation factor: as a condition for the inclusion of suppressor variable(s) in regression analysis. Open J Stat.

[45] Rothman K (2002). Epidemiology: an introduction.

[46] Bulled N (2022). A new approach to measurind the sinergy in a syndemic: Revising the SAVA syndemic among urban MSM in the United States. Glob Public Health.

[47] Knol MJ, van der tweel I, Grobbee DE, Numans ME, Geerlings MI (2007). Estimating interaction on an additive scale between continuous determinants in a logistic regression model. Int J Epidemiol..

[48] Stoicescu C, Ameilia R, Irwanto, Praptoraharjo I, Mahanani M. Syndemic and synergistic effects of intimate partner violence, crystal methamphetamine, and depression on HIV sexual risk behaviors among women who inject drugs in Indonesia. J Urban Heal. 2019;96:477–96. 10.1007/s11524-019-00352-6. 10.1007/s11524-019-00352-6 PMC6565664 30874946

[49] de Oliveira Gomes M, Castro R, Corrêa da Mota J, De Boni RB. Association of syndemic conditions and quality of life among people living with HIV/AIDS. AIDS Care*.* 2022:1–10. 10.1080/09540121.2022.2080801. 10.1080/09540121.2022.2080801 35621316

[50] Parsons JT, Antebi-Gruszka N, Millar BM, Cain D, Gurung S (2018). Syndemic conditions, HIV transmission risk behavior, and transactional sex among transgender women. AIDS Behav.

[51] Martínez O, Brady KA, Levine E, Page KR, Zea MC, Yamanis TJ, et al. Using syndemic theory to examine HIV sexual risk among latinx men who have sex with men in Philadelphia, PA: findings from the National HIV Behavioral Surveillance. EQHUIDAD*.* 2020;(13):217–36. 10.15257/ehquidad.2020.0009. 10.15257/ehquidad.2020.0009 PMC7039620 32095789

[52] Andersson GZ, Reinius M, Eriksson LE, Svedhem V, Mazi Esfahani F, Deuba K (2020). Stigma reduction interventions in people living with HIV to improve health-related quality of life. Lancet HIV.

[53] Schönnesson LN (2002). Psychological and existential issues and quality of life in people living with HIV infection. AIDS Care.

[54] Becker JT, Lopez OL, Dew MA, Aizenstein HJ (2004). Prevalence of cognitive disorders differs as a function of age in HIV virus infection. AIDS.

[55] Patel SS, Müller-Oehring EM, DeVaughn S, Fama R, Brönte-Stewart H, Poston K (2021). The effects of mood and cognition on daily functioning and quality of life in older people living with HIV and people with Parkinson's disease. Neuropsychology.

[56] Grant I (2008). Neurocognitive disturbances in HIV. Int Rev Psychiatry.

[57] Dòmenech-Abella J, Lara E, Rubio-Valera M, Olaya B, Moneta M, Rico-Uribe L (2017). Loneliness and depression in the elderly: the role of social network. Soc Psychiatry Psychiatr Epidemiol.

[58] Corrigan PW, Watson AC (2002). The paradox of self-stigma and mental illness. Clin Psychol Sci Pract.

[59] Mak WWS, Cheung RYM, Law RW, Woo J, Li PCK, Chung RWY (2007). Examining attribution model of self-stigma on social support and psychological well-being among people with HIV+/AIDS. Soc Sci Med.

[60] Balderson BH, Grothaus L, Harrison RG, McCoy K, Mahoney C, Catz S (2013). Chronic illness burden and quality of life in an aging HIV population. AIDS Care.

[61] Bayes-Marin I, Egea-Cortés L, Palacio-Vieira J, Bruguera A, Mesías-Gazmuri J, Llibre JM (2023). Determinants of depressive symptoms in people living with HIV: Findings from a population-based study with a gender perspective. Int J Environ Res Public Health.

[62] Shedlin MG, Decena CU, Oliver-Velez D. Initial acculturation and HIV risk among new hispanic immigrants. J Natl Med Assoc*.* 2005;97(7 SUPPL):32S-37S. PMID: 16080455. PMCID: PMC2640649. PMC2640649 16080455

[63] Betancur MN, Lins L, Oliveira IR, Brites C (2017). Quality of life, anxiety and depression in patients with HIV/AIDS who present poor adherence to antiretroviral therapy: a cross-sectional study in Salvador Brazil. Braz J Infect Dis.

[64] Dalessandro M, Conti CM, Gambi F, Falasca K, Doyle R, Conti P (2007). Antidepressant therapy can improve adherence to antiretroviral regimens among HIV-infected and depressed patients. J Clin Psychopharmacol.

[65] Springer SA, Dushaj A, Azar MM (2012). The impact of DSM-IV mental disorders on adherence to combination antiretroviral therapy among adult persons living with HIV/AIDS: a systematic review. AIDS Behav.

[66] Ettenhofer ML, Foley J, Castellon SA, Hinkin CH (2010). Reciprocal prediction of medication adherence and neurocognition in HIV/AIDS. Neurology.

[67] Chapman L, Westfall A, Modi R, Golin C, Keruly J, Quinilvan E (2020). HIV-related stigma, depression, and social support are associated with health-related quality of life among patients newly entering HIV care. AIDS Care.

[68] Ellis RJ, Rosario D, Clifford DB, McArthur JC, Simpson D, Alexander T (2010). Continued high prevalence and adverse clinical impact of human immunodeficiency virus - associated sensory neurophaty in the era of conbination antiretroviral therapy: the CHARTER study. Arch Neurol.

[69] Mayo NE, Brouillette MJ, Scott SC, Harris M, Smaill F, Smith G (2020). Relationships between cognition, function, and quality of life among HIV+ Canadian men. Qual Life Res.

[70] Greene M, Hessol NA, Perissinotto C, Zepf R, Hutton A, Foreman C (2018). Loneliness in older adults living with HIV. AIDS Behav.

[71] Alford K, Daley S, Banerjee S, Vera JH (2021). Quality of life in people living with HIV associated neurocognitive disorder: a scoping review study. PLoS One..

[72] Tozzi V, Costa M, Sampaolesi A, Fantoni M, Noto P, Ippolito G (2003). Neurocognitive performance and quality of life in patients with HIV infection. AIDS Res Hum Retroviruses.

[73] Leserman J (2003). The effects of stressful life events, coping, and cortisol on HIV infection. CNS Spectr.

[74] Jones D, Owens M, Kumar M, Cook R, Weiss SM (2014). The effect of relaxation interventions on cortisol levels in HIV-seropositive women. J Int Assoc Provid AIDS Care.

[75] Pellowski JA, Kalichman SC, Matthews KA, Adler N (2013). A pandemic of the poor: social disadvantage and the U.S. HIV epidemic. Am Psychol..

[76] Kang E, Delzell DAP, McNamara PE, Cuffey J, Cherian A, Matthew S (2016). Poverty indicators and mental health functioning among adults living with HIV in Delhi India. AIDS Care.

[77] Dray-Spira R, Gueguen A, Ravaud JF, Lert F (2007). Socioeconomic differences in the impact of HIV infection on workforce participation in France in the era of highly active antiretroviral therapy. Am J Public Health.

[78] Tsai AC, Bangsberg DR, Weiser SD (2013). Harnessing poverty alleviation to reduce the stigma of HIV in Sub-Saharan Africa. PLoS Med..

[79] Mayston R, Kinyanda E, Chishinga N, Prince M, Patel V (2012). Mental disorder and the outcome of HIV/AIDS in low-income and middle-income countries: a systematic review. AIDS.

[80] Han S, Hu Y, Wang L, Pei Y, Zhu Z, Qi X (2021). Perceived discrimination and mental health symptoms among persons living with HIV in China: the mediating role of social isolation and loneliness. J Assoc Nurses AIDS Care.

[81] Hatcher A, Lemus Hofstedler L, Doria K, Dworkin S, Weke E, Conroy A (2020). Mechanisms and perceived mental health changes after a livelihood intervention for HIV-positive Kenyans: Longitudinal, qualitative findings. Transcult Psychiatry.

[82] Chillag K, Guest G, Bunce A, Johnson L, Kilmarx PH, Smith DK (2006). Talking about sex in Botswana: social desirability bias and possible implications for HIV-prevention research. Afr J AIDS Res.

[83] Little RJ, Rubin DB (2000). Causal effects in clinical and epidemiological studies via potential outcomes: concepts and analytical approaches. Annu Rev Public Health.

