# Correction: Syndemic conditions and quality of life in the PISCIS Cohort of people living with HIV in Catalonia and the Balearic Islands: a cross sectional study

**DOI:** 10.1186/s12955-023-02139-5

**Published:** 2023-05-30

**Authors:** Jocelyn Mesías-Gazmuri, Cinta Folch, Jorge Palacio-Vieira, Andreu Bruguera, Laia Egea-Cortés, Carlos G. Forero, Juan Hernández, José M. Miró, Jordi Navarro, Melchor Riera, Joaquim Peraire, Lucía Alonso-García, Yesika Díaz, Jordi Casabona, Juliana Reyes-Urueña

**Affiliations:** 1 Centre of Epidemiological Studies of HIV/AIDS and STI of Catalonia (CEEISCAT), Health Department, Generalitat de Catalunya, Badalona, Spain; 2 Germans Trias I Pujol Research Institute (IGTP), Campus Can Ruti, Badalona, Spain; 3 PhD in Methodology of Biomedical Research and Public Health, Department of Pediatrics, Obstetrics and Gynecology, Preventive Medicine and Public Health, Universidad Autonoma de Barcelona, Badalona, Spain; 4 CIBER Epidemiología Y Salud Pública (CIBERESP), Madrid, Spain; 5 Department of Medicine. School of Medicine and Health Sciences, Universitat Internacional de Catalunya, Sant Cugat, Spain; 6 Grupo de Trabajo Sobre Tratamientos del VIH (gTt), Barcelona, Spain; 7 Infectious Diseases Service. Hospital Clínic-IDIBAPS. University of Barcelona, Barcelona, Spain; 8 CIBERINFEC, Instituto de Salud Carlos III, Madrid, Spain; 9 Infectious Diseases Department. Hospital, Universitari Vall d’Hebron, Barcelona, Spain; 10 Institut de Recerca Vall d’Hebron, Barcelona, Spain; 11 Hospital Son Espases, Palma de Mallorca, Spain; 12 Hospital Joan XXIII, IISPV, Universitat Rovira I Virgili, Tarragona, Spain; 13 Department of Pediatrics, Obstetrics and Gynecology and Preventive Medicine, Univ Autonoma de Barcelona, Badalona, Spain

**Correction: Health Qual Life Outcomes 21, 42 (2023)**

**https://doi.org/10.1186/s12955-023-02120-2**

The original article [1] contained an erroneous attribution of affiliation to Joaquim Peraire which has since been amended.
